# Tutoring in problem-based learning medical curricula: the influence of tutor background and style on effectiveness

**DOI:** 10.1186/1472-6920-5-20

**Published:** 2005-06-07

**Authors:** Michele Groves, Patricia Régo, Peter O'Rourke

**Affiliations:** 1 School of Medicine, University of Queensland, Herston Road, Qld 4006. Australia; 2 School of Population Health, University of Queensland, Herston Qld 4006. Australia

## Abstract

**Background:**

Evidence for the superiority of particular characteristics in PBL tutors in medical curricula is generally inconclusive. Most studies have investigated the effectiveness of content experts compared with that of non-experts as measured either by student satisfaction or academic achievement. A few have compared academic staff tutors with student tutors. The purpose of this study was to investigate the relationship between students' perception of overall tutor effectiveness, particular tutor behaviours, clinical qualifications and academic appointment.

**Method:**

A questionnaire designed to evaluate particular aspects of PBL tutoring technique, related either to subject-matter knowledge or to process-facilitation skill, as well as overall effectiveness, was distributed to students in first year of a PBL medical program at the end of each of three tutor terms. A total of 76 tutor terms were included in the study. Data analysis compared clinical with non-clinical tutors, and staff with non-staff tutors.

**Results:**

Clinically qualified tutors used their subject-matter knowledge significantly more than non-clinical tutors and were seen as being more empathic with their students. Staff tutors placed more emphasis on assessment than non-staff tutors and were seen as having greater skill in establishing and maintaining an environment of cooperation within their PBL groups than non-staff tutors.

**Conclusion:**

These results suggest that both subject-matter knowledge and process-facilitation skills are necessary but not individually sufficient characteristics of effective tutors.

## Background

Although problem-based learning has been at the forefront of reforms to medical curricula since its inception more than thirty years ago, conclusive evidence about its effectiveness as an educational approach remains elusive. In addition to research-related issues, such as non-randomised groups and small sample sizes, are variables inherent in the implementation of PBL curricula. These include variability in selection criteria for prospective students and the precise model of PBL employed [1]. An associated issue for which definitive answers have yet to be found relates to the qualifications and backgrounds of staff employed as facilitators in PBL tutorials.

The function of the tutor in PBL differs considerably from that of the tutor in conventional tutorials in which the tutor assumes a comparatively didactic role. A major feature of PBL is that learning is student-centred in that students take responsibility for identifying and addressing their own learning needs; tutors are required to facilitate this rather than adopt the position of content expert. Facilitation requires understanding of the learning process and primarily involves monitoring of student learning and promotion of effective group function. The student-centred learning approach of PBL means that for tutors, content knowledge should be subordinate to proficiency in group facilitation [2]. Thus, effective tutors promote student learning by creating a supportive environment which encourages active participation by all members of the group, by monitoring the quality of learning through questions and feedback and by encouraging the development of students' metacognitive skills [3].

Most of the studies [4-8] that have looked at the characteristics of skilled PBL tutors have compared the effectiveness of content experts with that of non-experts as measured either by student satisfaction or academic achievement. (In medical curricula, the problems that students address are presented as integrated clinical scenarios. Thus, content experts are seen as those having relevant subject matter knowledge. This refers not only to clinical skills such as history taking and physical examination but also to knowledge of basic science, public health and ethico-medico-legal issues as required by the problem. Thus, although non-clinically qualified academic staff may be seen as having expertise in the teaching/facilitation process and/or particular aspects of the curriculum, when it comes to subject matter knowledge, it is clinicians who are considered as experts.) Although findings from these studies are inconclusive, overall there is a slight preponderance in favour of content expertise over group facilitation skill with regard to both academic achievement and student satisfaction [4,6-8]. This is not a surprising outcome given the subjective nature of student perceptions of tutor effectiveness, possible conflict between students' perception of effective tutors and those characteristics essential to PBL (such as student-centredness, a non-didactic approach, emphasis on group function and self-directed learning) and the large number of factors that influence student academic achievement in addition to the skill of their PBL tutor. Findings from research into the relative effectiveness of staff versus student tutors are also inconclusive [9-11]. Additionally, Schmidt and Moust [12] in a study of PBL in a series of six-week health science courses looked at the influence of tutor style on student learning behaviour and academic achievement. Their findings suggested that the most effective tutors, as judged by the students, were those with both content knowledge and the ability to empathise with their students' circumstances. As far as we are aware, there have been no studies which have specifically explored the relationship between students' perception of overall effectiveness, particular tutor behaviours, clinical qualifications and academic appointment.

The medical program at the University of Queensland is a four-year, graduate entry program and features a PBL curriculum. The first year of the MBBS has an enrolment of approximately 270 students, divided into 26 PBL groups, and employs a range of PBL tutors over three teaching terms of about 11 weeks each. Although tutors may have medical, basic science or educational qualifications, the majority have expertise in one or other of the basic sciences, reflecting the dominant focus of the First Year curriculum. Tutors may be full-time academic staff or postgraduate students and others employed on a casual basis. All tutors are specifically trained in PBL before appointment to a student group and may teach up to three terms each year.

As stated above, tutoring in PBL tutoring has two components: facilitation skill and content knowledge. It may be expected that students would consider the principal strength of clinically qualified tutors to be their greater relevant content knowledge. In contrast, the principal strength of non-clinically qualified academic staff to the PBL process would be the facilitation skills derived from (often extensive) teaching experience. This study therefore has two aims. First, to compare the tutoring style and overall effectiveness (as perceived by their students) of Year 1 PBL tutors based on academic qualifications and appointment category; and second, to determine which of six specific tutor styles contributed most to students' perception of effective tutoring. The following hypotheses were tested: a) clinicians would be seen as more effective tutors than non-clinicians; b) full-time academic staff would be seen as more effective tutors than non-staff (casually-employed) tutors; and c) students would view subject matter knowledge as a more important determinant of effective tutoring than group facilitation skill. Thus, it is hoped that this study will provide a useful contribution to the PBL literature specifically by allowing both PBL tutors and their students to increase their understanding of the PBL process and how it may be optimally applied in medical education.

## Method

### Subjects

Subjects were the PBL tutors, each facilitating one of 26 groups (ten students per group) for an 11-week term in first year of the MBBS Program at the University of Queensland. Forty-two tutors were employed over the three terms of the teaching year, with each teaching an average of 1.90 groups. All tutors were given the opportunity to participate in this study and 40 agreed to evaluation by their students. Of the tutors, 26 were basic scientists, eight were medically qualified and six were classified as "other" having a social sciences background, predominantly in education. Additionally, 14 held full-time academic staff appointments (two clinicians, seven basic scientists and five "others") and 26 held non-staff appointments of which 14 were employed on a casual basis (five clinicians, 8 basic scientists and one teacher) and 12 were basic science PhD students, one of whom was also medically qualified. Thus, tutors were classified on the basis of both qualifications (clinical versus non-clinical) and type of appointment (staff versus non-staff)

Student participation was also voluntary. Students were allocated to PBL groups following enrolment and remained in the same group for the whole of First Year. Allocation was not random but was structured to ensure minimum variation between groups with regard to age, gender, academic background and nationality. Because the year consisted of three, eleven-week terms, each PBL group experienced three different tutors and thus provided three sets of data each. The total number of first year students was 270 allocated to 26 groups making the maximum number of student responses 810. However, because not all tutors agreed to participate, total of 702 student responses from 76 PBL tutor evaluations was received (overall response rate of 86.7%).

### Instrument

The questionnaire used was developed and validated at Maastricht University [10]. This instrument was chosen because it looks at specific tutor characteristics important to the PBL tutorial process. It consists of 39 items which explore tutoring style with respect to Knowledge of Subject Matter and Skill in Facilitation using a Likert-type rating scale. Within each of these categories are three scales which assess corresponding sub-categories of behaviour. The resultant six scales are:

#### (a) Knowledge of Subject Matter

• Use of expertise (UE): the degree to which the tutor uses his/her knowledge of relevant subject matter to help students (e.g. "The tutor used his/her subject-matter knowledge to help us").

• Cognitive congruence (CC): the degree to which the tutor is able to understand, and express him/herself at the students' level of knowledge (e.g. "The tutor had no difficulty understanding the group's problems with the subject-matter").

• Test orientation (TO): the degree to which the tutor focuses on summative assessment to direct the students' learning (e.g. "The tutor mentioned subjects we certainly had to know for the assessment of this course").

#### (b) Facilitation Skill

• Authority (AU): the degree to which a tutor uses his/her authority to direct students' activities within the group (e.g. "S/He intervened in ways that disturbed the progress of the group discussion").

• Role congruence (RC): the degree to which a tutor is able to empathise with, and relate to, students' lives (e.g. "The tutor understood the problems first-years have with their study").

• Cooperation orientation (CO): the degree to which a tutor is interested in encouraging cooperation among members of the group (e.g. "At regular intervals, the tutor evaluated with us the group's functioning").

The items were randomised in the order in which they occurred in the questionnaire except for the last item which asked students to rate how well the tutor played his/her role overall. This last item was thus considered to be a measure of the students' perception of their tutor's *overall *effectiveness.

### Analysis

Participating tutors were evaluated by each of the students in their PBL groups at the end of each teaching term, resulting in a maximum of 20 evaluations per tutor per term depending on the number of PBL groups they facilitated. Completed responses were scanned and collated into a spreadsheet containing data on each tutor's qualifications and type of appointment. Although no effect due to tutor age and gender was expected, this information was also included to establish if age and gender exerted a confounding effect on the results. The data were analysed using the SPSS^® ^program [13]. The various items for each outcome variable were described by Schmidt & Moust [12], However principal components analysis was used to confirm the correct loading onto each of the items into the appropriate outcome variable (use of expertise, cognitive congruence, test orientation, authority, role congruence, cooperation orientation and overall effectiveness, as perceived by the students). These outcome variables formed the basis of all subsequent data analysis.

Of the explanatory variables, age was transformed into a categorical variable: 20–39 years and 40+ years. The other categorical variables were gender (M or F), qualifications (clinical, basic science or other) and appointment type (staff, student or casual). Initial analysis indicated that within the latter two variables, the only significant differences were between clinicians compared to non-clinicians and between staff compared to non-staff. Consequently, these variables were recoded (as clinician or other, and staff or non-staff) for all subsequent analyses. Finally, because students were allocated to PBL groups in a way that ensured that each group contained a similar mix of students with regard to age, gender, academic and cultural background, there was relatively little variation between PBL groups compared to the variation between students within each group. Hence, the PBL group for each term was regarded as the experimental unit and each group's mean scores rather than individual responses were used for all analyses.

The reliability of each of the scales and of the items was calculated using Cronbach's alpha coefficient of internal consistency. For this type of analysis, an alpha coefficient >0.70 indicates acceptable reliability.

Univariate analysis was performed for each outcome variable using one-way ANOVA to assess the relationship between categorical and outcome variables. *p*-values <0.05 were considered to indicate a statistically significant difference. Subsequently, general linear modelling was used to analyse the influence of characteristics which univariate analysis had shown to have a significant relationship with tutoring style. The final model was selected by backwards elimination until all remaining terms were significant. The model was then screened for interactions between these terms. As no interactions were significant, and adjustment for other terms in the final model was minimal, results are presented for the one-way ANOVA for each explanatory variable.

## Results

A summary of the analysis of the data is given in Table 1. This indicates that the reliability of the instrument varied between scales from 0.55 (Authority) to 0.95 (Use of Expertise). Of the six scales measured, only Authority (0.55) and Role Congruence (0.57) fell below 0.70 while the reliability of the instrument, over all 39 items, was 0.92 which is acceptable.

**Table 1:** Evaluation of the reliability, correlation with perceived overall effectiveness, mean and standard deviation of each of the measured scales

		**UE ** Max = 36	**CC ** Max = 28	**TO ** Max = 16	**AU ** Max = 20	**RC ** Max = 24	**CO ** Max = 24	**Overall Effectiveness ** Max = 4
**Reliability**		0.92	0.95	0.85	0.55	0.57	0.91	0.70

**Correlation with Overall Effectiveness**	Correlation	0.79	0.67	0.52	-0.06	0.77	0.69	
	p-value	**<0.01**	**<0.01**	**<0.01**	0.63	**<0.01**	**<0.01**	

**Corrected Model**^1^	Grand mean^2^	24.9	21.2	11.2	13.4	18.0	20.8	3.5
	Standard Deviation	3.1	2.7	1.4	2.3	2.4	2.4	0.6

^1 ^Corrected for attenuation

^2 ^The mean of all scores in all groups

Correlation coefficients were calculated to determine the strength of the association between students' perception of their tutor's overall effectiveness and each of the tutor behaviours assessed. With the exception of the scale measuring use of authority, all scales showed a significant, although variable, association with overall effectiveness score. Use of expertise and role congruence correlated most strongly. When the multivariate relationship between individual scales and overall score was analysed, there was a close association between five scales (Use of Expertise, Cognitive Congruence, Test Orientation, Role Congruence and Cooperation Orientation). A final regression model retained only Use of Expertise (β-coefficient = 0.53, p <0.01), Authority (β-coefficient = -0.20, p <0.01) and Role Congruence (β-coefficient = 0.39, p <0.01).

Table 2 summarises the results of the univariate analysis of tutor characteristics and compares the average scores for each scale based on gender, age academic qualifications and appointment type. These results indicate that there was no significant difference between male and female tutors on any of the scales despite a consistent trend for males to have higher scores. With regard to

**Table 2:** Comparison of scale mean scores on the basis of gender, age, academic qualifications and appointment type.

**Outcome variable**	**Scale**	**UE**	**CC**	**TO**	**AU**	**RC**	**CO**	**Overall Effectiveness**
**Gender**	Female N = 57	23.3	20.5	10.8	13.0	17.4	20.2	3.4
	Male N = 19	24.9	20.9	11.4	13.4	17.8	20.8	3.5
	p-value	0.07	0.56	0.09	0.47	0.56	0.33	0.28

**Age**	20–39 N = 23	23.1	20.1	10.4	12.7	17.6	20.1	3.5
	40+ N = 53	24.0	20.8	11.2	13.2	17.5	20.4	3.4
	p-value	0.26	0.31	**0.03**	0.41	0.84	0.67	0.44

**Qualifications**	Clinical N = 15	25.9	21.5	11.3	13.7	18.6	20.9	3.6
	Other N = 61	23.2	20.4	10.8	12.9	17.2	20.2	3.4
	p-value	**<0.01**	0.19	0.30	0.21	**0.05**	0.27	0.21

**Appointment**	Casual N = 51	23.3	20.2	10.7	13.0	17.4	19.9	3.3
	Staff N = 25	24.5	21.4	11.4	13.3	17.8	21.1	3.5
	p-value	0.17	0.09	**0.03**	0.61	0.53	**0.04**	0.22

• Age: Older tutors tended to have higher scores on all scales except Role Congruence. However, this difference only reached statistical significance for Test Orientation (p = 0.03), which suggests that older tutors emphasise assessment as a means of encouraging group learning more than younger tutors.

• Qualifications: Clinicians tended to have higher scores on all scales than non-clinicians but these were only significant for Use of Expertise (p < 0.01) and Role Congruence (p = 0.05).

• Appointment: There was a tendency for staff tutors to have higher scores on all scales than non-staff tutors but these only reached statistical significance for Test Orientation (p = 0.03) and Cooperation Orientation (p = 0.04). That is, staff tutors focussed more clearly than non-staff tutors on assessment to motivate learning and were better able to promote effective group function.

## Discussion and Conclusion

Full-time academic staff are more likely than non-staff (casual and postgraduate student) tutors both to emphasise summative assessment as a factor in learning, and to display greater skill in establishing and maintaining a team approach among their students. These findings may be due to the considerably greater time that faculty spend in teaching and assessment tasks in general, as well as experience in dealing with student issues relative to casual tutors and postgraduate students. Although age was shown not to have a significant impact on any of these behaviours, it is worth noting that most (85.7%) staff tutors were in the 40+ age group, compared to 53.8% for non-staff tutors.

Using the same instrument, Moust and Schmidt [10] investigated differences in tutoring style between staff and non-staff tutors. They found that staff tutors scored significantly lower than non-staff tutors in three of the six behaviours: cognitive congruence, test orientation and role congruence, with staff tutors scoring higher on the authority scale only. This is in contrast to our findings which found significant differences in only two of the scales (and those in favour of staff tutors), test orientation and cooperation orientation. The discrepancy between the two studies may be explained, at least in part, by the different composition of the non-staff group between the two studies. The non-staff group in our study was more heterogeneous, consisting of both casual and student tutors, thereby blurring the distinction between staff and non-staff, particularly with regard to age – only 8.3% of student tutors were over 40 years compared with 85.7% of casual tutors.

With regard to clinical versus non-clinical tutors, medically-qualified PBL tutors are significantly more likely to make use of their expertise in facilitating their groups' learning and to empathise with their students' lives as medical students. This is not surprising, given their own prior experience as medical students themselves and that they can be assumed to also have a large body of clinically-relevant knowledge which they are able to apply to addressing the problem being studied.

None of these findings are reflected in the students' ratings for the last item in the questionnaire: how well they perceived that their tutor played his/her role (overall effectiveness). This may be due to the relative crudity of the four-point scale for each item in the questionnaire. Such an effect would be particularly noticeable with regard to the item on overall effectiveness which has a maximum score of only four points, as well as a relatively higher standard deviation than those for the other scales. Additionally, when taken in conjunction with the finding of significant correlations between five of the six behaviours and perceived effectiveness, it may be that characteristics other than those measured in this study also contribute to the students' perception of their tutor's effectiveness.

Nevertheless, the lack of a significant difference between clinicians and non-clinicians in students' ratings for overall effectiveness, given that they rate clinicians' use of expertise significantly higher, is striking. This is especially so in view of the substantial correlation between use of expertise and overall effectiveness, and raises the question of what exactly is meant by the term "expertise" in relation to problem-based learning. The issue of whether content experts are more effective as PBL tutors than those without content expertise has been, and continues to be, a subject of debate in the literature with many studies finding in favour of the experts [4,7]. In research into PBL in integrated medical curricula, the experts are assumed to be clinicians, with basic scientists and others designated as non-experts for the reasons discussed earlier. However, it is possible that, in First Year of the MBBS where the principal emphasis is on the basic sciences, expertise in the basic sciences is seen as an equally valuable characteristic in PBL tutors as clinical expertise. In contrast, the Second-Year curriculum has a much greater clinical focus and the majority of PBL tutors are medically qualified. A follow-up study of tutors in Year 2 is planned.

An alternative explanation is that it is the way a tutor uses his/her expertise, rather than the degree of expertise, which is important in determining a tutor's score on this scale. That is, it is possible that clinicians adopt a tutor-centred, rather than student-centred approach to PBL tutoring but that this characteristic is not regarded by students as having a major impact on their tutor's effectiveness. Indeed, a recent study has shown that clinicians tend to ask questions directly of students, whereas non-clinicians are more likely to encourage students to ask questions of each other [6].

The same argument can be applied to the finding regarding staff versus non-staff tutors. That is, that although full-time staff are more test-oriented and better at establishing a collaborative environment for student learning, these differences are not enough in themselves to impact significantly on overall effectiveness, again suggesting that characteristics other than those measured at least partially influence this assessment.

In summary, these findings suggest that, although clinicians and staff tutors consistently scored higher on each of the measured behaviours than did non-clinicians and non-staff, most of these differences are not statistically significant and do not have a substantial impact on students' assessment of their effectiveness as PBL tutors. This conclusion has implications for the recruitment and training of PBL tutors in that it suggests that both subject matter knowledge and process facilitation skills are necessary but not individually sufficient characteristics of effective tutors. Developing a broad range of strategies to encourage optimal group functioning and to stimulate student learning should therefore be a major focus of tutor preparation.

## Competing Interests

The author(s) declare that they have no competing interests.

## Authors' Contributions

MG conceived the study, participated in its design and coordination, contributed to the analyses and drafted the manuscript. PR participated in the study design and coordination, carried out the statistical analysis and approved the final draft. PO'R carried out the statistical analysis and approved the final draft of the manuscript.

## Pre-publication history

The pre-publication history for this paper can be accessed here:



## References

[B1] Vernon DT, Blake RL (1993). Does problem-based learning work? A meta-analysis of evaluative research. Academic Medicine.

[B2] Barrows HS, Tamblyn RM (1980). Problem-Based Learning: An Approach to Medical Education.

[B3] Maudsley G (1999). Roles and responsibilities of the problem based learning tutor in the undergraduate medical curriculum. BMJ.

[B4] Davis WK, Nairn R, Paine ME, Anderson RM, Oh MS (1992). Effects of expert and non-expert facilitators on the small-group process and on student performance. Acad Med.

[B5] Dolmans DH, Gijselaers WH, Moust JH, de Grave WS, Wolfhagen IH, van der Vleuten CP (2002). Trends in research on the tutor in problem-based learning: conclusions and implications for educational practice and research. Med Teach.

[B6] Gilkison A (2003). Techniques used by 'expert' and 'non-expert' tutors to facilitate problem-based learning tutorials in an undergraduate medical curriculum. Med Educ.

[B7] Hendry GD, Huy P, Lyon PM, Gordon J (2002). Student evaluation of expert and non-expert problem-based learning tutors. Medical Teacher.

[B8] Schmidt HG, van der Arend A, Moust JH, Kokx I, Boon L (1993). Influence of tutors' subject-matter expertise on student effort and achievement in problem-based learning. Acad Med.

[B9] Moust JHC, de Volder ML, Nuy HJP (1989). Peer teaching and higher level cognitive learning outcomes in problem-based learning. Higher Education.

[B10] Moust JHC, Schmidt HG (1995). Facilitating small-group learning: a comparison of student and staff tutors' behaviour. Instructional Science.

[B11] de Grave WS, de Volder ML, Gijselaers WH, Damoiseaux V, Z.N.Nooman HGSESE (1990). Peer teaching and problem-based learning: Tutor characteristics, tutor functioning, group functioning and student achievement. Innovation in medical education: An evaluation of its present status.

[B12] Schmidt HG, Moust JHC (1995). What makes a tutor effective? A structural-equations modelling approach to learning in problem-based curricula. Academic Medicine.

[B13] (2002). SPSS 11.5 for Windows.

